# Impact of β-blockers on in-hospital mortality in patients with heart failure: a retrospective propensity-score matched analysis based on MIMIC-IV database

**DOI:** 10.3389/fphar.2024.1448015

**Published:** 2024-08-13

**Authors:** Xin Wang, Yuzhu Zhang, Jiangling Xia, Hongyu Xu, Lu Zhang, Nianhai Feng, Xiaona An

**Affiliations:** ^1^ Department of Interventional Vascular, Zibo 148 Hospital, China RongTong Medical Healthcare Group Co., Ltd., Zibo, Shandong, China; ^2^ Department of Anesthesiology, Zibo Central Hospital, Zibo, Shandong, China

**Keywords:** β-blockers, mortality, heart failure, propensity score matching, los (length of stay) hospital, mimic

## Abstract

**Introduction:**

This study assessed the relationship between β-blockers treatment and in-hospital mortality among individuals diagnosed with heart failure (HF).

**Methods:**

A retrospective cohort study was carried out on 9,968 HF patients sourced from the Medical Information Mart for Intensive Care (MIMIC)-IV database. Propensity score matching (PSM) was employed to balance the baseline differences. A multivariate regression analysis was utilized to evaluate the impact of β-blockers therapy on in-hospital mortality.

**Results:**

Among the 9,968 patients, 6,439 (64.6%) were β-blockers users. Before matching, the overall in-hospital mortality rate was 12.2% (1,217/9,968). Following PSM, a total of 3,212 patient pairs were successfully matched. The analysis revealed a correlation between β-blockers therapy and decreased in-hospital mortality (odds ratio 0.51 [0.43–0.60], *P* < 0.001), as well as shorter Los (length of stay) hospital (β −1.43 [−1.96∼−0.09], *P* < 0.001). Notably, long-acting β-blockers treatment was linked to a decreased risk of in-hospital mortality (odds ratio 0.55 [0.46–0.65], *P* < 0.001) and a shorter Los hospital (β −1.21 [−1.80∼−0.63], *P* < 0.001). Conversely, the research results did not show a notable decrease in-hospital mortality (odds ratio 0.66 [0.44–1.01], *P* = 0.051) or Los hospital (β −1.01 [−2.2∼−0.25], *P* = 0.117) associated with short-acting β-blocker therapy.

**Discussion:**

β-blockers therapy in the intensive care unit demonstrates potential benefits in lowering the risk of in-hospital mortality and reducing the duration of hospitalization among patients with HF. Specifically, long-acting β-blockers exhibit a protective effect by significantly decreasing both in-hospital mortality and Los hospital. Conversely, the study did not observe a substantial impact on in-hospital mortality or Los hospital duration in this cohort of patients following the administration of short-acting β-blockers.

## 1 Introduction

Heart failure (HF) is a multifaceted and enduring clinical condition resulting from structural or functional abnormalities in the myocardium that impede the heart’s ability to effectively circulate blood (Yancy et al., 2013; Heidenreich et al., 2022). Despite the introduction of numerous new medications for heart failure in recent times, patients with HF still face high rates of morbidity and mortality (Njoroge and Teerlink, 2021). Historically, β-blockers were considered contraindicated for heart failure treatment due to their negative effects on myocardial contractility (Foody et al., 2002). However, with the emergence of the neurohormonal hypothesis of heart failure (HF), there has been a growing interest in exploring the potential therapeutic role of β-blockers in treating heart failure patients. According to the European Society of Cardiology (ESC) guidelines, the use of β-blockers is suggested for all individuals diagnosed with symptomatic HF unless contraindications are present (Dickstein et al., 2008).

Over activation of the renin-angiotensin-aldosterone system (RAAS) and the sympathetic nervous system (SNS) is a key of HF (Eskerud et al., 2019; Schwinger, 2021). The SNS signaling pathway leads to a β-adrenoceptor mediated increase renin release, retention of salt and water by the kidney, constriction of peripheral arteries and increased myocardial contractility via activation of β1-adrenoceptors, in an attempt to maintain adequate cardiac output (Hartupee and Mann, 2017). Increased activation of the β-adrenoceptor in the HF increases myocardial energy and oxygen demand and oxidative stress, aggravating heart failure (Santulli and Iaccarino, 2016). β-blockers, specifically the selective β1-blockers, have been shown to have a favorable effect on heart failure because they prevent catecholamine release and sympathetic activity, diminish heart rate and improve diastolic function (Kubon et al., 2011).

β-blockers are commonly prescribed for various cardiovascular conditions, such as HF (Satwani et al., 2004; Kubon et al., 2011) and myocardial infarction (Kjekshus, 1986; Cucherat, 2007), owing to their ability to decrease sympathetic neural activity and regulate heart rate (HR). Nevertheless, the impact of β-blockers therapy on clinical outcomes in heart failure remains uncertain. Previous investigations primarily focused on short-acting β-blockers like esmolol in tachycardic patients due to their perceived safety compared to β-blockers (Kakihana et al., 2020; Hasegawa et al., 2021). A systematic review reported that β-blockers could lower heart failure patients’ mortality risk and hospitalization (Shibata et al., 2001). However, these studies are limited by small sample sizes and notable diversity in patient baseline characteristics, leading to inconsistent results. Furthermore, research has predominantly centered on short-acting β-blockers (esmolol or landiolol) in septic patients with tachycardia leaving out studies on long-acting β-blockers. A study showed that long-acting β-blocker therapy may have a protective role in patients with sepsis, reducing the 28-day and 90-day mortality. However, short-acting β-blocker (esmolol) treatment did not reduce the mortality in sepsis (Ge et al., 2023).

Therefore, this study used propensity score matching (PSM) to examine the correlation between β-blockers and in-hospital mortality among heart failure patients and investigated whether long-acting or short-acting β-blockers are linked to improved clinical outcomes in this patient population.

## 2 Materials and methods

### 2.1 Data selection

Data for this retrospective cohort study was extracted from the Medical Information Mart for Intensive Care (MIMIC)-IV database (version 2.2), a publicly available repository of intensive care information in the United States. The dataset comprises information on patients admitted to the ICU at Beth Israel Deaconess Medical Center in Boston, Massachusetts, from 2008 to 2019 (Johnson et al., 2016). Access to the database was obtained after the author, Xiaona An, completed the Collaborative Institutional Training Initiative exam (Certification number: 60,273,273) and conducted the data extraction process. Due to the anonymized nature of the data, informed consent was deemed unnecessary.

### 2.2 Study population and data extraction

All individuals admitted to the intensive care unit (ICU) with a primary diagnosis of heart failure were included in the study from the MIMIC-IV database (Xia et al., 2023). Exclusion criteria involved individuals under 18 years old, those with incomplete data, and patients discharged or deceased within 48 h of ICU admission. Participants were divided into two cohorts based on their use of β-blockers: the intervention group receiving β-blockers and the control group not receiving β-blockers.

Data collection involved querying the PostgreSQL (version 14.2) database utilizing Structured Query Language (SQL) to obtain baseline characteristics. The information encompassed patient demographics (gender, age), vital signs (HR - heart rate, MAP - mean arterial pressure, SpO2 - oxygenated hemoglobin saturation), laboratory test results (HCT - hematocrit, HGB - hemoglobin, PLT - platelets, WBC - white blood cell count, anion gap, BUN - blood urea nitrogen, calcium, creatinine, glucose, sodium, potassium), comorbidities (diabetes, MI - myocardial infarction, PVD - peripheral vascular disease, dementia, cerebrovascular disease, chronic pulmonary disease, renal disease, cancer), and severity at admission assessed by the Charlson Comorbidity Index (CCI), Simplified Acute Physiology Score II (SAPS II), and Oxford Acute Severity of Illness Score (OASIS).

### 2.3 Outcome

The primary focus of the research was on in-hospital mortality rates, with the date of death for each patient obtained from the Social Security Death Index records provided by the US government. The date of death was required to precede the date of hospital discharge to ensure accurate and valid results (Zhang and Shi, 2021). The secondary outcome under investigation was the hospital length of stay (LOS).

### 2.4 Statistics analysis

Patient characteristics were described collectively and by specific groups (β-blocker exposed and non-β-blocker exposed). Continuous variables were presented as mean or median, depending on the data distribution, with analyses conducted using *ANOVA* or *Kruskal–Wallis H-test* based on normality. Categorical variables were reported as percentages and evaluated through Chi-square tests.

PSM was conducted to balance the baseline differences with a caliper width of 0.2 logits of the standard difference (Morgan, 2018). Patients were matched in a 1:1 ratio through a nearest-neighbor approach, ensuring each individual in the β-blockers group was paired with a corresponding individual in the non-β-blocker group. The effectiveness of the PSM process was examined utilizing the SMD, where an SMD of ≤0.1 indicated a satisfactory balance for baseline propensity models.

R software (http://www.R-project.org, The R Foundation) and Free Statistics software version 1.9.1 were utilized to perform statistical analyses. Statistical significance was considered at a *P*-value < 0.05.

## 3 Results

### 3.1 Baseline characteristics

**FIGURE 1:**
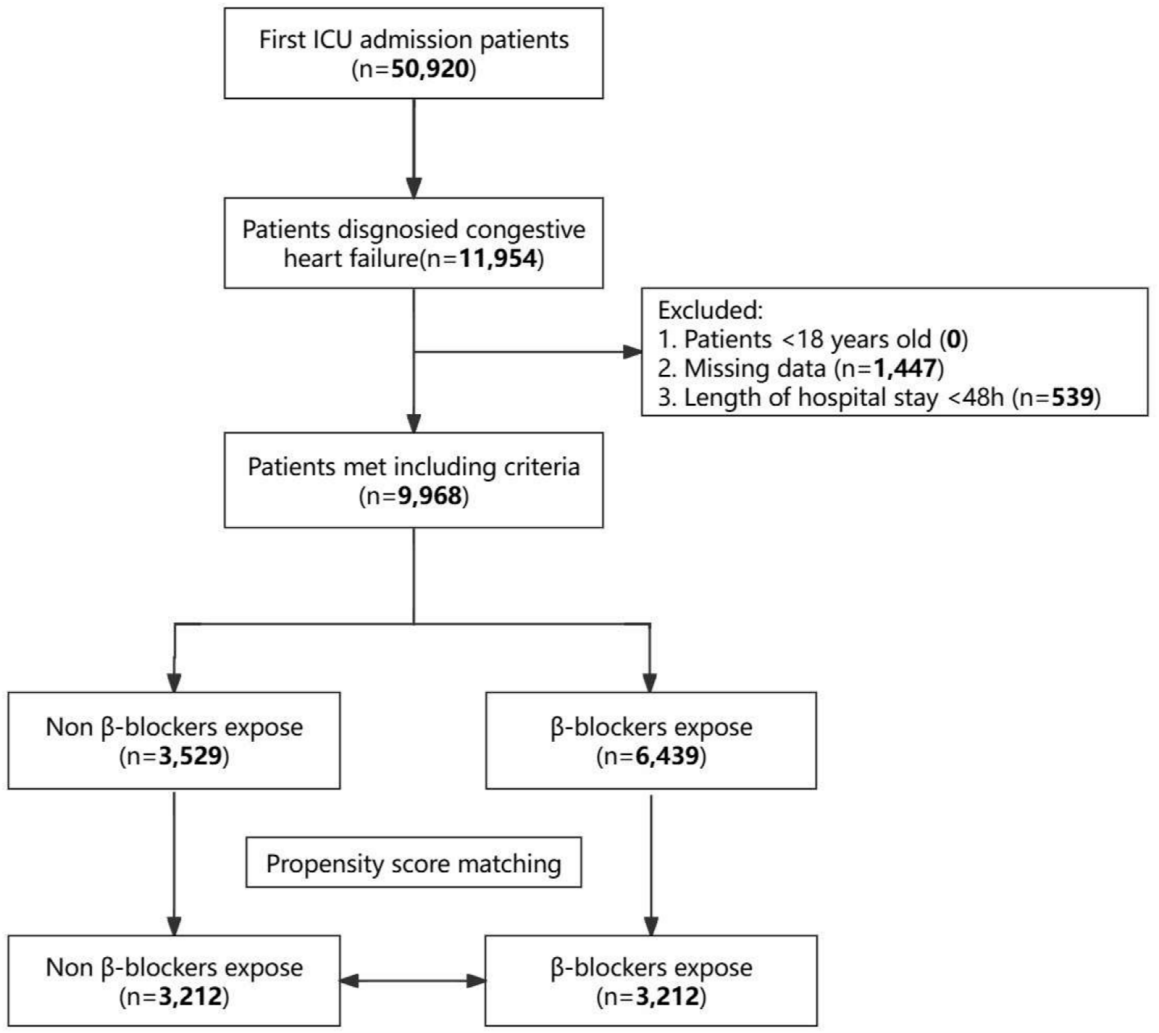
Flowchart of cohort selection.

Among the initial 50,920 individuals admitted to the ICU and extracted from the MIMIC-IV database, 11,954 were diagnosed with heart failure. After applying exclusion criteria, 9,968 patients were selected for this study cohort. Among these, 6,439 individuals (64.6%) received β-blockers post-ICU admission, while 3,529 patients (35.4%) did not. The selection process is illustrated in Figure 1. Supplementary Table S1 details the classification of the different β-blockers utilized in the study. Regarding the patients who received β-blockers following ICU admission, the most commonly prescribed was metoprolol (5,111 of 6,439 [79.38%]), followed by carvedilol (654 of 6,439 [10.16%]), labetalol (391 of 6,439 [6.07%]), atenolol (131 of 6,439 [2.03%]), esmolol (95 of 6,439 [1.48%]), propranolol (25 of 6,439 [0.39%]), nadolol (21 of 6,439 [0.33%]), betaxolol (6 of 6,439 [0.09%]), bisoprolol (4 of 6,439 [0.06%]), and nebivolol (1 of 6,439 [0.01%]).

Table 1 presents the baseline characteristics of the patients before PSM. The cohort’s median age was 73.5 ± 13.7 years, with male patients accounting for 55.4%. The overall incidence of in-hospital mortality was 12.2% (1,217/9,968). A comparison between patients who received β-blockers therapy and those who did not revealed that individuals on β-blockers were generally older (73.8 ± 13.3 vs 72.8 ± 14.4, *P* < 0.001), had a higher heart rate (84.7 ± 16.0 vs 83.1 ± 16.6, *P* < 0.001), and a higher MAP (81.2 ± 11.1 vs 77.3 ± 10.3, *P* < 0.001). Furthermore, there were noticeable discrepancies in disease severity between the two groups, as illustrated by the SAPS II scores (37.8 ± 11.9 vs 41.8 ± 14.3, *P* < 0.001) and OASIS (32.1 ± 8.8 vs 33.9 ± 9.6, *P* < 0.001) scores. However, the groups had no significant difference in the Charlson Comorbidity Index (CCI) score (7.5 ± 2.5 vs 7.4 ± 2.6, *P* = 0.423). Patients who received β-blockers therapy had lower in-hospital mortality (559 of 6,439 [8.7%] vs 658 of 3,529 [18.6%], *P* < 0.001), shorter Los hospital (8.0 [5.1, 12.9] vs 9.2 [5.6, 15.3], *P* < 0.001), and shorter Los ICU (2.2 [1.3, 4.0] vs 3.0 [1.6, 5.8], *P* < 0.001) compared with the non-β-blockers group.

**TABLE 1:** Baseline characteristics of the study participants.

Variables	All participants (n = 9,968)	Non β-blockers (n = 3,529)	β-blockers (n = 6,439)	*p*-value
Gender (male %)	5,520 (55.4)	1895 (53.7)	3,625 (56.3)	0.013
Age (year)	73.5 ± 13.7	72.8 ± 14.4	73.8 ± 13.3	<0.001
HR (beats/min)	84.1 ± 16.2	83.1 ± 16.6	84.7 ± 16.0	<0.001
MAP (mmHg)	79.8 ± 10.9	77.3 ± 10.3	81.2 ± 11.1	<0.001
SpO_2_	96.5 ± 2.2	96.4 ± 2.5	96.6 ± 1.9	<0.001
HCT (%)	30.8 ± 6.7	30.3 ± 6.9	31.0 ± 6.5	<0.001
HGB (g/L)	10.1 ± 2.2	9.8 ± 2.3	10.2 ± 2.2	<0.001
PLT (10^9/L)	177.0 (130.0.237.0)	172.0 (122.0, 235.0)	180.0 (133.0, 238.0)	<0.001
WBC (10^9/L)	12.1 (8.8, 16.8)	12.4 (8.6, 17.7)	12.0 (8.8, 16.3)	0.004
Anion gap (mmol/L)	16.9 ± 4.6	17.5 ± 5.1	16.7 ± 4.3	<0.001
BUN (mmol/L)	29.0 (19.0, 46.0)	32.0 (20.0, 52.0)	27.0 (19.0, 43.0)	<0.001
Calcium (mg/dL)	8.3 ± 0.8	8.2 ± 0.9	8.3 ± 0.8	<0.001
Creatinine (mg/dL)	1.3 (1.0, 2.1)	1.4 (1.0, 2.3)	1.3 (0.9, 1.9)	<0.001
Glucose (mg/dL)	122.0 ± 43.7	121.5 ± 45.0	122.3 ± 43.0	0.366
Sodium (mmol/L)	139.6 ± 4.9	139.5 ± 5.4	139.6 ± 4.6	0.239
Potassium (mmol/L)	4.7 ± 0.9	4.8 ± 0.9	4.7 ± 0.8	<0.001
Diabetes, n (%)	4,034 (40.5)	1,366 (38.7)	2,668 (41.4)	0.008
MI, n (%)	3,308 (33.2)	965 (27.3)	2,343 (36.4)	<0.001
PVD, n (%)	1,647 (16.5)	507 (14.4)	1,140 (17.7)	<0.001
Dementia, n (%)	469 (4.7)	184 (5.2)	285 (4.4)	0.076
Cerebrovascular disease, n (%)	1,367 (13.7)	387 (11)	980 (15.2)	<0.001
Chronic pulmonary disease, n (%)	3,680 (36.9)	1,378 (39)	2,302 (35.8)	0.001
Renal disease, n (%)	3,798 (38.1)	1,345 (38.1)	2,453 (38.1)	0.987
Cancer, n (%)	1,059 (10.6)	430 (12.2)	629 (9.8)	<0.001
CCI	7.5 ± 2.5	7.4 ± 2.6	7.5 ± 2.5	0.413
SAPS II	39.2 ± 12.9	41.8 ± 14.3	37.8 ± 11.9	<0.001
Oasis	32.7 ± 9.1	33.9 ± 9.6	32.1 ± 8.8	<0.001
In-hospital mortality, n (%)	1,217 (12.2)	658 (18.6)	559 (8.7)	<0.001
Los hospital (day)	8.4 (5.2, 13.8)	9.2 (5.6, 15.3)	8.0 (5.1, 12.9)	<0.001
Los ICU (day)	2.4 (1.3, 4.7)	3.0 (1.6, 5.8)	2.2 (1.3, 4.0)	<0.001

Data are presented as mean ± SD, medians [interquartile ranges] or numbers (percentages).

HR, heart rate; MAP, mean arterial pressure; SpO2, oxygenated hemoglobin saturation; HCT, hematocrit; HGB, hemoglobin; PLT, platelets; WBC, white blood cell count; BUN, blood urea nitrogen; MI, myocardial infarction; PVD: peripheral vascular disease; CCI, charlson comorbidity index, SAPS II, simplified acute physiology score; Oasis, Oxford Acute Severity of Illness Score; Los, length of stay.

After PSM, 3,212 patients who were not administered β-blockers were successfully paired with 3,212 patients who received β-blockers (Supplementary Table S2). Subsequent comparison of baseline characteristics among the two groups post-PSM revealed no statistical variances (*P* > 0.05) in most parameters, except for in-hospital mortality, Los hospital, and ICU (*P* > 0.05) (Supplementary Table S3).

### 3.2 Correlations between β-blockers exposure and outcomes

Univariate logistic and linear regression analyses were utilized to identify risk factors for in-hospital mortality (Supplementary Table S4) and Los hospital (Supplementary Table S5). Age, HR, MAP, SpO2, HCT, HGB, WBC, Anion gap, BUN, Calcium, Creatinine, PVD, Dementia, Cerebrovascular disease, Cancer, CCI, SAPS Ⅱ, and Oasis were correlated with increased hospital mortality risk and Los hospital.

Multivariate logistic and linear regression analyses were conducted to evaluate the impact of β-blockers therapy on in-hospital mortality and length of hospital stay while controlling for confounding variables. Variables with a significance level below 0.05 in the univariate analysis were included in the multivariate analysis (Table 2). The findings suggested a significant correlation between β-blockers therapy and a decreased risk of in-hospital mortality in both the pre-and post-PSM cohorts, with odds ratios of 0.51 (95% CI: 0.44–0.59, *P* < 0.001) and 0.51 (95% CI: 0.43–0.60, *P* < 0.001) respectively. Moreover, β-blockers therapy was linked to a shorter Los hospital in both the pre- and post-PSM cohorts, with β coefficients of −1.52 (95% CI: −1.95 to −1.09, *P* < 0.001) and −1.43 (95% CI: −1.96 to −0.09, *P* < 0.001), respectively.

**TABLE 2:** Association between β-blocker and In-hospital mortality, Los hospital in multiple regression model.

Variables	In-hospital mortality		Los hospital	
	Or 95%CI	*p-*value	β 95%CI	*p-*value
Pre-matched cohort				
Unadjusted	0.41 (0.37–0.47)	<0.001	−2.14 (−2.57∼-1.71)	<0.001
Model I	0.4 (0.36–0.46)	<0.001	−2.05 (−2.47∼-1.62)	<0.001
Model II	0.49 (0.43–0.56)	<0.001	−1.63 (−2.06∼-1.2)	<0.001
Model III	0.51 (0.44–0.59)	<0.001	−1.52 (−1.95∼-1.09)	<0.001
Post-matched cohort				
Unadjusted	0.57 (0.49–0.66)	<0.001	−1.39 (−1.94∼-0.83)	<0.001
Model I	0.57 (0.49–0.66)	<0.001	−1.42 (−1.97∼-0.87)	<0.001
Model II	0.56 (0.48–0.65)	<0.001	−1.36 (−1.90∼-0.83)	<0.001
Model III	0.51 (0.43–0.60)	<0.001	−1.43 (−1.96∼-0.90)	<0.001

OR, odds ratio; CI, confidence interval; Los, length of stay.

Model I = Adjusted for (age + gender). Model II = Model I + (HR + MAP + SpO_2_ + HCT + HGB + WBC + Anion gap + BUN + Calcium + Creatinine). Model III = Model II + (PVD + Dementia + Cerebrovascular disease + Cancer + CCI + APSIII + SOFA, score).

### 3.3 Correlation between short-acting β-blockers therapy and outcomes

Following PSM, a cohort of 539 patients who were administered short-acting β-blockers (specifically esmolol, carvedilol, and labetalol) were compared with a matched group of 539 patients who did not receive short-acting β-blockers. Baseline characteristics analysis post-PSM indicated no significant differences between the groups (Supplementary Table S6; Supplementary Figure S1; *P* > 0.05, SMD< 0.1). Multivariate logistic and linear regression analyses were conducted to evaluate the impact of short-acting β-blockers therapy on in-hospital mortality and Los hospital while adjusting for potential confounding factors. Variables with a significance level below 0.05 in the univariate analysis were included in the multivariate analysis (Table 3). The analysis revealed that short-acting β-blockers therapy did not significantly decrease in-hospital mortality, with an odds ratio of 0.66 (95% CI: 0.44–1.01, *P* = 0.051), nor did it significantly shorten Los hospital, with a β coefficient of −1.01 (95% CI: −2.27 to 0.25, *P* = 0.117).

**TABLE 3:** Association between short-acting β-blockers and In-hospital mortality, Los hospital in multiple regression model after PSM.

Variables	In-hospital mortality		Los hospital	
	Or 95%CI	*p-*value	β 95%CI	*p-*value
Unadjusted	0.70 (0.48–1.04)	0.078	−0.90 (−2.21–0.40)	0.175
Model I	0.69 (0.47–1.02)	0.065	−0.87 (−2.17–0.43)	0.191
Model II	0.67 (0.45–1.01)	0.053	−1.02 (−2.30–0.26)	0.119
Model III	0.66 (0.44–1.01)	0.051	−1.01 (−2.27–0.25)	0.117

OR, odds ratio; CI, confidence interval; Los, length of stay.

Model I = Adjusted for (age + gender). Model II = Model Ⅰ + (HR + MAP + SpO_2_ + HCT + HGB + WBC + Anion gap + BUN + Calcium + Creatinine). Model III = Model Ⅱ + (PVD + Dementia + Cerebrovascular disease + Cancer + CCI + APSIII + SOFA, score).

### 3.4 Correlation between long-acting β-blockers therapy and outcomes

Following PSM, 2,662 patients who were administered long-acting β-blockers (including metoprolol, atenolol, nadolol, propranolol, betaxolol, bisoprolol, nebivolol) were compared with an equal number of patients who did not receive such treatment. Baseline characteristics revealed no statistically significant differences between the groups following PSM (Supplementary Table S7; Supplementary Figure S2; *P* > 0.05, SMD< 0.1). Subsequent multivariate logistic and linear regression analyses were performed to evaluate the impact of long-acting β-blockers therapy on in-hospital mortality and Los hospital while adjusting for confounding variables. Variables with a significance level below 0.05 in the univariate analysis were included in the multivariate analysis (Table 4). The findings revealed a significant correlation between long-acting β-blockers treatment and a reduction in in-hospital mortality, showing an odds ratio of 0.55 (95% CI: 0.46–0.65, *P* < 0.001). Furthermore, patients in the long-acting β-blockers group exhibited a shorter Los hospital, as evidenced by a β coefficient of −1.21 (−1.80 to −0.63, *P* < 0.001).

**TABLE 4:** Association between long-acting β-blockers and In-hospital mortality, Los hospital in multiple regression model after PSM.

Variables	In-hospital mortality		Los hospital	
	Or 95%CI	*p-*value	β 95%CI	*p-*value
Unadjusted	0.60 (0.51–0.70)	<0.001	−1.25 (−1.86∼-0.64)	<0.001
Model I	0.59 (0.51–0.70)	<0.001	−1.23 (−1.84∼-0.62)	<0.001
Model II	0.57 (0.48–0.68)	<0.001	−1.23 (−1.83∼-0.64)	<0.001
Model III	0.55 (0.46–0.65)	<0.001	−1.21 (−1.80∼-0.63)	<0.001

OR, odds ratio; CI, confidence interval; Los, length of stay.

Model I = Adjusted for (age + gender). Model II = Model Ⅰ + (HR + MAP + SpO_2_ + HCT + HGB + WBC + Anion gap + BUN + Calcium + Creatinine). Model III = Model II + (PVD + Dementia + Cerebrovascular disease + Cancer + CCI + APSIII + SOFA, score).

### 3.5 Subgroup analyses

**FIGURE 2:**
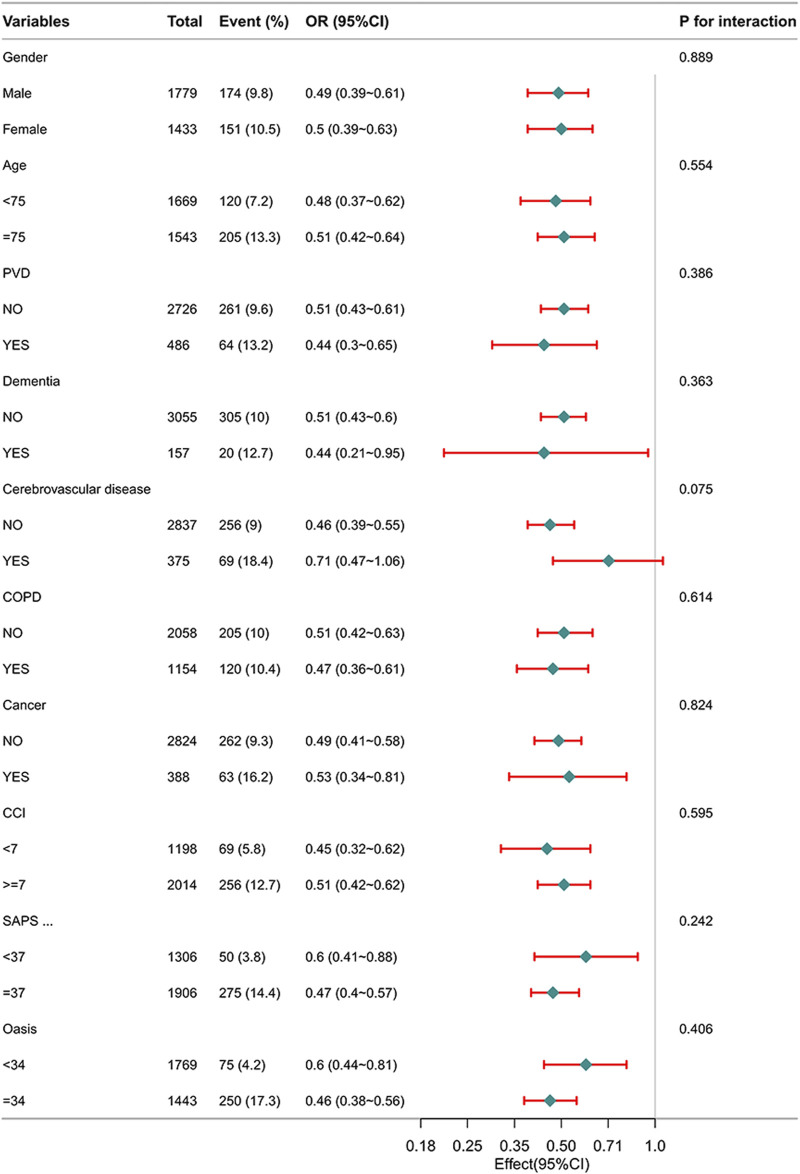
Subgroup analysis of the relationship between β-blockers therapy and in-hospital mortality in patients with HF.

Figure 2 illustrates the correlation between β-blockers therapy and in-hospital mortality among different subgroups. The *P* -values for interactions among these subgroups indicate no notable influence on in-hospital mortality. Subgroup analyses have been appropriately adjusted for the variables within the PSM to ensure the validity of the results.

## 4 Discussion

In our retrospective cohort study, we discovered that variables such as Age, HR, MAP, SpO_2_, HCT, HGB, WBC, Anion gap, BUN, Calcium, Creatinine, PVD, Dementia, Cerebrovascular disease, Cancer, CCI, SAPS Ⅱ, and Oasis were linked to an increased risk of hospital mortality and Los hospital. Patients who were administered β-blockers exhibited a notable decrease in the likelihood of in-hospital mortality. Notably, the utilization of extended-release β-blockers was correlated with enhanced in-hospital survival rates for individuals with heart failure. Conversely, there was no observed decrease in mortality among heart failure patients receiving short-acting β-blockers therapy.

Epidemiological studies have consistently highlighted the potential benefits of β-blockers as an independent favorable factor in reducing in-hospital mortality among patients with sepsis. For example, a study (Yang et al., 2023) revealed a notable relationship between the administration of β-blockers and a reduction in in-hospital mortality among patients with sepsis. Morelli et al. conducted a pioneering randomized controlled trial to assess the effectiveness and safety of esmolol in treating septic shock (Morelli et al., 2013), demonstrating that β-blockers benefit patient prognosis (Kubon et al., 2011; Ge et al., 2023). Additionally, two studies indicated that the esmolol infusion lowered 28-day mortality rates and improved cardiovascular function in septic shock patients (Xinqiang et al., 2015; Morelli et al., 2016). However, some studies did not show statistically significant variances in 28-day mortality rates between sepsis or septic shock patients who were treated with short-acting β-blockers and those in the control group (Wang et al., 2017; Kakihana et al., 2020). A meta-analysis revealed that ultra-short-acting β-blockers were linked to improved 28-day mortality in sepsis patients with persistent tachycardia following initial resuscitation (Shibata et al., 2001). While multiple studies have demonstrated the potential of β-blockers in reducing mortality among sepsis patients, research on their impact on patients with heart failure remains limited. Most of these studies were constrained by small sample sizes and variations in baseline characteristics, potentially causing bias in the results. Our consistent findings revealed a positive correlation between the administration of β-blocker therapy and decreased in-hospital mortality among ICU patients with heart failure, even after adjusting for potential confounders. We implemented the PSM method to minimize selection bias and ensured a balanced distribution of baseline covariates between the two groups. Additionally, we employed multivariate Cox regression analysis to alleviate potential confounding biases. Our study included 539 patients treated with short-acting β-blockers and an equal number who were not. Contrary to the findings of the afore-mentioned meta-analysis, our results indicated that short-acting β-blocker treatment did not significantly improve in-hospital mortality post-PSM. Discrepancies in baseline patient characteristics and sample sizes may partly account for this inconsistency.

The relationship between long-acting β-blockers and patient mortality in the context of heart failure remains a topic requiring further investigation. A review (Kakihana et al., 2020) published in 2019 suggested a potential link between pre-sepsis β-blocker exposure and decreased mortality, yet the available data were insufficient for meta-analysis. To better elucidate the correlation between long-acting β-blockers and mortality in HF patients, we included 2,662 patients in the long-acting β-blockers group following PSM, ensuring a balanced distribution of baseline characteristics. Our results aligned with previous investigations, demonstrating that long-acting β-blockers therapy was correlated with a notable reduction in patient mortality rates.

Furthermore, through subgroup analyses following PSM, we observed a positive impact of β-blockers treatment on in-hospital mortality among HF patients. The increased *P*-value for interaction in the subgroup analyses enhanced the reliability of our findings.

β-blockers, specifically the selective β1 blockers, have been shown to have a favorable effect on heart failure because they prevent catecholamine release and sympathetic activity, diminish heart rate and improve diastolic function (Kubon et al., 2011). Increasing evidence indicates that high heart rate predicts poorer prognosis and adequate heart rate control may help improve outcomes in managing heart failure, so the role of β-blockers might be more important than ever. Treatment with β-blockers for sepsis to relieve the sympathetic stress response has been previously investigated. Therefore, we conducted a retrospective study to determine the association between the use of β-blockers and mortality in patients with sepsis to better guide clinical management.

This study also presents several limitations. Firstly, being a retrospective study, it cannot definitively establish a causal correlation between β-blockers and the risk of in-hospital mortality in the heart failure population. Secondly, despite the use of PSM to address confounding variables, the presence of residual confounders cannot be completely ruled out. Thirdly, we did not include information on the specific dosages of β-blockers utilized in the study, leaving the effects of dosage on mortality unclear. Finally, most patients of this study received metoprolol. Future investigations should aim to address these limitations to validate and further elaborate on our results. Therefore, it is advised that the outcomes of our study be interpreted with caution.

## 5 Conclusion

Our retrospective analysis demonstrated a significant correlation between β-blocker therapy in the ICU and a reduced incidence of in-hospital mortality among individuals with heart failure. Notably, long-acting β-blocker treatment appeared to offer a protective benefit to these patients, while the administration of short-acting β-blockers did not demonstrate a substantial impact on mortality rates. The findings need to be validated in future large-scale randomized controlled trials on different populations or additional outcomes. Finally, a prospective randomized controlled double-blind clinical trial is urgently needed to evaluate the potential benefit and safety of β-blockers given in various doses and routes in heart failure patients to further prove the results.

## Data Availability

The raw data supporting the conclusions of this article will be made available by the authors, without undue reservation.
