# Supplementary material for: Impact of β-blockers on in-hospital mortality in patients with heart failure: a retrospective propensity-score matched analysis based on MIMIC-IV database
Source: Front Pharmacol. 2024 Aug 13;15:1448015. doi: 10.3389/fphar.2024.1448015 (PMC11347275; doi:10.3389/fphar.2024.1448015)
Supplement: Supplementary file 1 [file DataSheet1.docx]

Supplementary Material

# Supplementary Figures and Tables

## Supplementary Figures


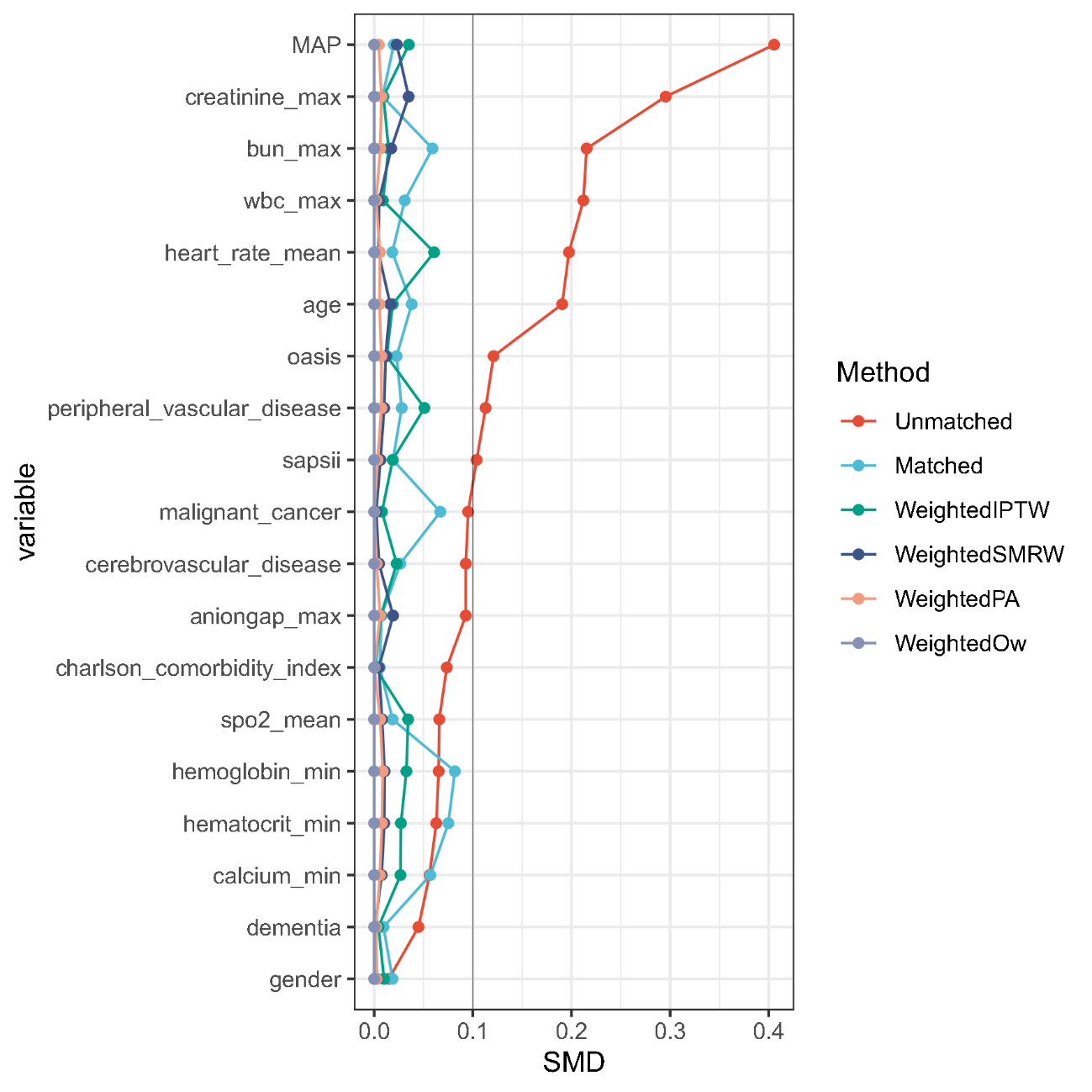


**Supplementary Figure 1.** Standardized mean difference of variables before and after propensity score matching for short-acting β-blockers. SMD, Standardized mean difference; MAP, mean arterial pressure; bun, blood urea nitrogen; wbc, white blood cell count.

**
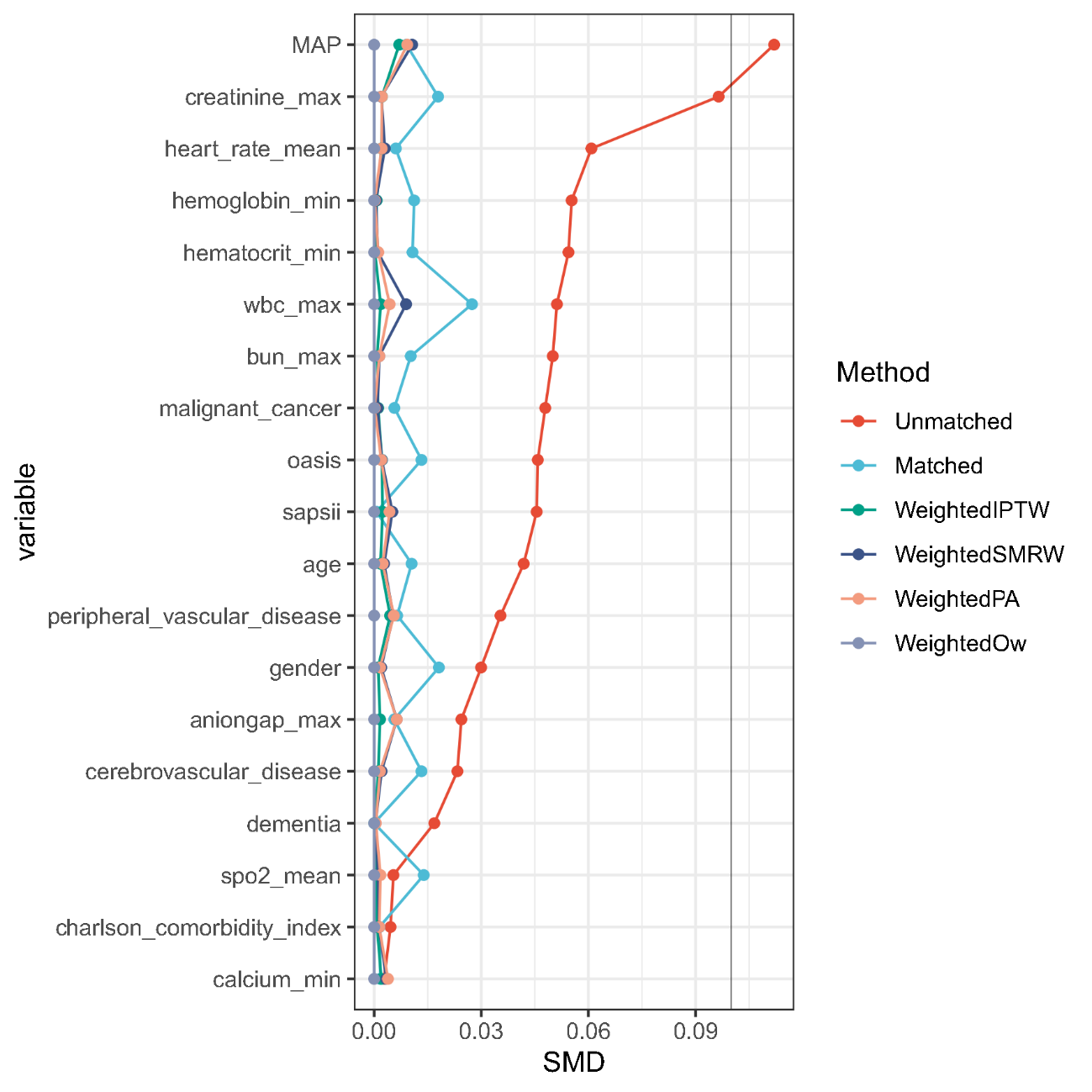
**

**Supplementary Figure 2.** Standardized mean difference of variables before and after propensity score matching for long-acting β-blockers. SMD, Standardized mean difference; MAP, mean arterial pressure; bun, blood urea nitrogen; wbc, white blood cell count.

## 1.2 Supplementary Tables

## Table S1. Details regarding the classification of β-blockers

| **β-blockers** | **Patients (%)** |
| --- | --- |
| Atenolol | 131/6,439 (2.03%) |
| Betaxolol | 6/6,439 (0.09%) |
| Bisoprolol | 4/6,439 (0.06%) |
| Carvedilol | 654/6,439 (10.16%) |
| Esmolol | 95/6,439 (1.48%) |
| Labetalol | 391/6,439 (6.07%) |
| Metoprolol | 5,111/6,439 (79.38%) |
| Nadolol | 21/6,439 (0.33%) |
| Nebivolol | 1/6,439 (0.01) |
| Propranolol | 25/6,439 (0.39%) |

## Table S2. Imbalance of patient characteristics before and after PSM in the assessment of In-hospital mortality.

| **Variables** | **Unmatched** | | | | **Matched** | | | |
| --- | --- | --- | --- | --- | --- | --- | --- | --- |
|  | **Non**  **β-blockers** | **β-blockers** | **SMD** | **SMD** | **Non**  **β-blockers** | **β-blockers** | **SMD** | **SMD** |
|  | **3529** | **6439** |  | **0.1** | **3212** | **3212** |  | **0.1** |
| Gender | 1634 (46.3) | 2814 (43.7) | 0.052 | <0.1 | 1472 (45.8) | 1433 (44.6) | 0.024 | <0.1 |
| Age | 72.77 (14.36) | 73.85 (13.26) | 0.078 | <0.1 | 73.14 (14.12) | 72.86 (13.43) | 0.021 | <0.1 |
| HR | 83.10 (16.58) | 84.70 (16.02) | 0.098 | <0.1 | 83.43 (16.48) | 83.39 (15.92) | 0.002 | <0.1 |
| MAP | 77.32 (10.27) | 81.18 (11.07) | 0.361 | >0.1 | 78.01 (10.29) | 78.32 (10.00) | 0.031 | <0.1 |
| SpO2 | 96.42 (2.54) | 96.60 (1.94) | 0.08 | <0.1 | 96.54 (2.16) | 96.57 (2.05) | 0.015 | <0.1 |
| HCT | 30.35 (6.94) | 31.03 (6.53) | 0.101 | >0.1 | 30.42 (6.89) | 30.64 (6.52) | 0.033 | <0.1 |
| HGB | 9.85 (2.29) | 10.18 (2.21) | 0.149 | >0.1 | 9.90 (2.28) | 9.97 (2.19) | 0.033 | <0.1 |
| WBC | 14.64 (11.01) | 13.77 (9.74) | 0.084 | <0.1 | 14.41 (10.71) | 14.32 (9.00) | 0.009 | <0.1 |
| Anion gap | 17.47 (5.12) | 16.66 (4.29) | 0.173 | >0.1 | 17.08 (4.74) | 17.10 (4.68) | 0.006 | <0.1 |
| BUN | 40.37 (28.60) | 34.83 (24.07) | 0.209 | >0.1 | 38.12 (26.26) | 38.74 (27.03) | 0.023 | <0.1 |
| Calcium | 8.16 (0.87) | 8.31 (0.76) | 0.181 | >0.1 | 8.20 (0.84) | 8.22 (0.78) | 0.02 | <0.1 |
| Creatinine | 2.05 (1.84) | 1.81 (1.74) | 0.135 | >0.1 | 1.95 (1.77) | 1.99 (1.87) | 0.022 | <0.1 |
| PVD | 507 (14.4) | 1140 (17.7) | 0.091 | <0.1 | 483 (15.0) | 486 (15.1) | 0.003 | <0.1 |
| Dementia | 184 (5.2) | 285 (4.4) | 0.037 | <0.1 | 155 (4.8) | 157 (4.9) | 0.003 | <0.1 |
| Cerebrovascular disease | 387 (11.0) | 980 (15.2) | 0.126 | >0.1 | 367 (11.4) | 375 (11.7) | 0.008 | <0.1 |
| Cancer | 430 (12.2) | 629 (9.8) | 0.077 | <0.1 | 369 (11.5) | 388 (12.1) | 0.018 | <0.1 |
| CCI | 7.45 (2.57) | 7.49 (2.47) | 0.017 | <0.1 | 7.43 (2.54) | 7.48 (2.54) | 0.019 | <0.1 |
| SAPS Ⅱ | 41.80 (14.28) | 37.81 (11.91) | 0.304 | >0.1 | 40.35 (13.26) | 40.50 (12.94) | 0.012 | <0.1 |
| Oasis | 33.94 (9.59) | 32.07 (8.82) | 0.203 | >0.1 | 33.24 (9.29) | 33.30 (9.25) | 0.006 | <0.1 |

An absolute MSD of <10% was considered to support the assumption of a balance between the groups. SMD, standardized mean differences. Data are presented as mean [SD] or as numbers (percentages).

## Table S3. Baseline characteristics of patients on admission after PSM

| **Variables** | **All participants**  **(n = 6424)** | **Non β-blockers**  **(n = 3212)** | **β-blockers**  **(n = 3212)** | ***P*-value** |
| --- | --- | --- | --- | --- |
| Gender (male %) | 3519 (54.8) | 1740 (54.2) | 1779 (55.4) | 0.328 |
| Age (year) | 73.0 ± 13.8 | 73.1 ± 14.1 | 72.9 ± 13.4 | 0.409 |
| HR (beats/min) | 83.4 ± 16.2 | 83.4 ± 16.5 | 83.4 ± 15.9 | 0.938 |
| MAP (mmHg) | 78.2 ± 10.1 | 78.0 ± 10.3 | 78.3 ± 10.0 | 0.217 |
| SpO2 | 96.6 ± 2.1 | 96.5 ± 2.2 | 96.6 ± 2.1 | 0.558 |
| HCT (%) | 30.5 ± 6.7 | 30.4 ± 6.9 | 30.6 ± 6.5 | 0.183 |
| HGB (g/L) | 9.9 ± 2.2 | 9.9 ± 2.3 | 10.0 ± 2.2 | 0.181 |
| PLT (10^9/L) | 177.0 (128.0, 237.0) | 174.0 (124.8, 236.0) | 180.0 (131.0, 238.0) | 0.006 |
| WBC (10^9/L) | 12.4 (8.8, 17.3) | 12.2 (8.5, 17.4) | 12.4 (9.2, 17.2) | 0.052 |
| Anion gap (mmol/L) | 17.1 ± 4.7 | 17.1 ± 4.7 | 17.1 ± 4.7 | 0.82 |
| BUN (mmol/L) | 30.0 (20.0, 49.0) | 30.0 (20.0, 49.0) | 30.0 (20.0, 49.0) | 0.509 |
| Calcium (mg/dL) | 8.2 ± 0.8 | 8.2 ± 0.8 | 8.2 ± 0.8 | 0.434 |
| Creatinine (mg/dL)) | 1.4 (1.0, 2.2) | 1.4 (1.0, 2.2) | 1.4 (1.0, 2.2) | 0.359 |
| Glucose (mg/dL) | 121.3 ± 43.4 | 121.9 ± 44.5 | 120.7 ± 42.4 | 0.242 |
| Sodium (mmol/L) | 139.5 ± 4.9 | 139.4 ± 5.2 | 139.6 ± 4.7 | 0.124 |
| Potassium (mmol/L) | 4.7 ± 0.9 | 4.7 ± 0.9 | 4.7 ± 0.9 | 0.195 |
| Diabetes, n (%) | 2563 (39.9) | 1235 (38.4) | 1328 (41.3) | 0.018 |
| MI, n (%) | 2051 (31.9) | 885 (27.6) | 1166 (36.3) | < 0.001 |
| PVD, n (%) | 969 (15.1) | 483 (15) | 486 (15.1) | 0.917 |
| Dementia, n (%) | 312 (4.9) | 155 (4.8) | 157 (4.9) | 0.908 |
| Cerebrovascular disease, n (%) | 742 (11.6) | 367 (11.4) | 375 (11.7) | 0.755 |
| Chronic pulmonary disease, n (%) | 2412 (37.5) | 1258 (39.2) | 1154 (35.9) | 0.007 |
| Renal disease, n (%) | 2473 (38.5) | 1199 (37.3) | 1274 (39.7) | 0.054 |
| Cancer, n (%) | 757 (11.8) | 369 (11.5) | 388 (12.1) | 0.462 |
| CCI | 7.5 ± 2.5 | 7.4 ± 2.5 | 7.5 ± 2.5 | 0.449 |
| SAPS Ⅱ | 40.4 ± 13.1 | 40.3 ± 13.3 | 40.5 ± 12.9 | 0.637 |
| Oasis | 33.3 ± 9.3 | 33.2 ± 9.3 | 33.3 ± 9.3 | 0.801 |
| In-hospital mortality, n (%) | 856 (13.3) | 531 (16.5) | 325 (10.1) | < 0.001 |
| Los hospital （day） | 8.7 (5.5, 14.1) | 9.1 (5.6, 14.9) | 8.4 (5.4, 13.7) | < 0.001 |
| Los ICU (day) | 2.6 (1.4, 5.0) | 2.9 (1.5, 5.4) | 2.3 (1.4, 4.2) | < 0.001 |

Abbreviations: HR, heart rate; MAP: Mean arterial pressure; HCT, hematocrit; HGB, hemoglobin; PLT, platelets; WBC, white blood cell count; BUN, blood urea nitrogen; MI, myocardial infarction; PVD: peripheral vascular disease; CCI: Charlson comorbidity index, SAPS II: Simplified Acute Physiology Score; Oasis: Oxford Acute Severity of Illness Score; Los: length of stay.

## Table S4. Univariate logistic regression analyses to identify the risks for in-hospital mortality

| **Variables** | **OR (95%CI)** | ***P*-value** |
| --- | --- | --- |
| Gender | 1.09 (0.96~1.23) | 0.177 |
| Age | 1.02 (1.02~1.03) | <0.001 |
| HR | 1.01 (1.01~1.02) | <0.001 |
| MAP | 0.97 (0.96~0.97) | <0.001 |
| SpO_2_ | 0.96 (0.94~0.99) | 0.003 |
| HCT | 0.98 (0.97~0.99) | <0.001 |
| HGB | 0.9 (0.88~0.93) | <0.001 |
| PLT | 1 (1~1) | 0.052 |
| WBC | 1.02 (1.02~1.03) | <0.001 |
| Anion gap | 1.09 (1.08~1.11) | <0.001 |
| BUN | 1.01 (1.01~1.02) | <0.001 |
| Calcium | 0.72 (0.67~0.77) | <0.001 |
| Creatinine | 1.1 (1.07~1.13) | <0.001 |
| Glucose | 1 (1~1) | 0.031 |
| Sodium | 1.01 (1~1.02) | 0.053 |
| Potassium | 1.22 (1.15~1.3) | <0.001 |
| Diabetes | 0.88 (0.77~0.99) | 0.037 |
| MI | 1.19 (1.05~1.35) | 0.006 |
| PVD | 1.23 (1.05~1.43) | 0.009 |
| Dementia | 1.44 (1.12~1.85) | 0.005 |
| Cerebrovascular disease | 1.78 (1.53~2.07) | <0.001 |
| Chronic pulmonary disease | 1.01 (0.9~1.15) | 0.814 |
| Renal disease | 1.27 (1.13~1.44) | <0.001 |
| Cancer | 1.75 (1.48~2.08) | <0.001 |
| CCI | 1.16 (1.13~1.19) | <0.001 |
| SAPS Ⅱ | 1.07 (1.06~1.07) | <0.001 |
| Oasis | 1.11 (1.1~1.12) | <0.001 |
| Los Hospital | 1.01 (1~1.01) | <0.001 |
| Los ICU | 1.07 (1.06~1.08) | <0.001 |
| β-blockers | 0.41 (0.37~0.47) | <0.001 |

Abbreviations: HR, heart rate; MAP: Mean arterial pressure; HCT, hematocrit; HGB, hemoglobin; PLT, platelets; WBC, white blood cell count; BUN, blood urea nitrogen; MI, myocardial infarction; PVD: peripheral vascular disease; CCI: Charlson comorbidity index, SAPS II: Simplified Acute Physiology Score; Oasis: Oxford Acute Severity of Illness Score; Los: length of stay.

## Table S5. Univariate linear regression analyses to identify the risks for Los hospital

| Variables | β (95%CI) | *P* |
| --- | --- | --- |
| Gender | -0.3 (-0.71,0.12) | 0.161 |
| Age | -0.09 (-0.11, -0.08) | < 0.001 |
| HR | 0.08 (0.07,0.09) | < 0.001 |
| MAP | -0.06 (-0.08, -0.04) | < 0.001 |
| SpO2 | 0.19 (0.1,0.29) | < 0.001 |
| HCT | -0.2 (-0.23, -0.17) | < 0.001 |
| HGB | -0.63 (-0.72, -0.54) | < 0.001 |
| PLT | -0.01 (-0.01,0) | < 0.001 |
| WBC | 0.08 (0.06,0.1) | < 0.001 |
| Anion gap | 0.08 (0.04,0.13) | < 0.001 |
| BUN | 0.02 (0.02,0.03) | < 0.001 |
| Calcium | -1.4 (-1.66, -1.15) | < 0.001 |
| Creatinine | 0.24 (0.12,0.36) | < 0.001 |
| Glucose | 0 (0,0) | 0.942 |
| Sodium | -0.08 (-0.13, -0.04) | < 0.001 |
| Potassium | 0.1 (-0.14,0.34) | 0.395 |
| Diabetes | 0.29 (-0.13,0.71) | 0.174 |
| MI | -0.28 (-0.72,0.16) | 0.207 |
| PVD | 0.74 (0.18,1.29) | 0.01 |
| Dementia | -1.15 (-2.12, -0.17) | 0.021 |
| Cerebrovascular disease | 1.55 (0.95,2.15) | < 0.001 |
| Chronic pulmonary disease | -0.12 (-0.54,0.31) | 0.596 |
| Renal disease | 0.34 (-0.08,0.77) | 0.116 |
| Cancer | 3.25 (2.58,3.92) | < 0.001 |
| CCI | 0.23 (0.15,0.31) | < 0.001 |
| SAPS Ⅱ | 0.12 (0.11,0.14) | < 0.001 |
| Oasis | 0.21 (0.19,0.24) | < 0.001 |
| In-hospital mortality | 1.16 (0.53,1.79) | < 0.001 |
| Los ICU | 0.9912 (0.957,1.0254) | < 0.001 |
| β-blockers | -2.14 (-2.57, -1.71) | < 0.001 |

Abbreviations: HR, heart rate; MAP: Mean arterial pressure; HCT, hematocrit; HGB, hemoglobin; PLT, platelets; WBC, white blood cell count; BUN, blood urea nitrogen; MI, myocardial infarction; PVD: peripheral vascular disease; CCI: Charlson comorbidity index, SAPS II: Simplified Acute Physiology Score; Oasis: Oxford Acute Severity of Illness Score; Los: length of stay.

## Table S6. Baseline characteristics of patients on admission after PSM (short-acting β-blockers)

| **Variables** | **All participants**  **(n = 1078)** | **Non short-acting BB**  **(n = 539)** | **short-acting BB**  **(n = 539)** | ***P*-value** |
| --- | --- | --- | --- | --- |
| Gender (male %) | 577 (53.5) | 286 (53.1) | 291 (54) | 0.76 |
| Age (year) | 70.7 ± 14.0 | 70.4 ± 13.8 | 71.0 ± 14.3 | 0.532 |
| HR (beats/min) | 80.6 ± 15.8 | 80.8 ± 15.9 | 80.5 ± 15.7 | 0.763 |
| MAP (mmHg) | 81.8 ± 11.7 | 81.9 ± 12.1 | 81.7 ± 11.3 | 0.743 |
| SpO_2_ | 96.6 ± 2.0 | 96.6 ± 2.1 | 96.7 ± 1.9 | 0.759 |
| HCT (%) | 30.5 ± 6.6 | 30.7 ± 6.7 | 30.2 ± 6.5 | 0.217 |
| HGB (g/L) | 9.9 ± 2.2 | 10.0 ± 2.2 | 9.8 ± 2.2 | 0.179 |
| PLT (10^9/L) | 180.0 (134.0, 239.0) | 179.0 (132.0, 245.5) | 182.0 (134.0, 236.0) | 0.656 |
| WBC (10^9/L) | 11.5 (8.4, 15.8) | 11.5 (8.4, 16.0) | 11.4 (8.4, 15.6) | 0.559 |
| Anion gap (mmol/L) | 17.4 ± 4.8 | 17.4 ± 5.0 | 17.3 ± 4.7 | 0.905 |
| BUN (mmol/L) | 33.0 (22.0, 54.0) | 33.0 (21.0, 54.5) | 33.0 (22.0, 54.0) | 0.198 |
| Calcium (mg/dL) | 8.3 ± 0.8 | 8.3 ± 0.8 | 8.3 ± 0.7 | 0.349 |
| Creatinine (mg/dL)) | 1.5 (1.1, 2.6) | 1.5 (1.0, 2.4) | 1.6 (1.1, 2.7) | 0.165 |
| Glucose (mg/dL) | 121.5 ± 47.5 | 119.9 ± 45.5 | 123.0 ± 49.4 | 0.289 |
| Sodium (mmol/L) | 139.5 ± 5.0 | 139.3 ± 5.0 | 139.7 ± 4.9 | 0.172 |
| Potassium (mmol/L) | 4.8 ± 0.9 | 4.8 ± 1.0 | 4.7 ± 0.9 | 0.211 |
| Diabetes, n (%) | 515 (47.8) | 245 (45.5) | 270 (50.1) | 0.127 |
| MI, n (%) | 356 (33.0) | 187 (34.7) | 169 (31.4) | 0.244 |
| PVD, n (%) | 214 (19.9) | 110 (20.4) | 104 (19.3) | 0.647 |
| Dementia, n (%) | 43 ( 4.0) | 21 (3.9) | 22 (4.1) | 0.876 |
| Cerebrovascular disease, n (%) | 153 (14.2) | 74 (13.7) | 79 (14.7) | 0.663 |
| Chronic pulmonary disease, n (%) | 403 (37.4) | 226 (41.9) | 177 (32.8) | 0.002 |
| Renal disease, n (%) | 510 (47.3) | 246 (45.6) | 264 (49) | 0.272 |
| Cancer, n (%) | 90 ( 8.3) | 40 (7.4) | 50 (9.3) | 0.271 |
| CCI | 7.6 ± 2.6 | 7.6 ± 2.6 | 7.6 ± 2.6 | 0.925 |
| SAPS Ⅱ | 39.2 ± 13.3 | 39.1 ± 13.7 | 39.3 ± 13.0 | 0.755 |
| Oasis | 32.2 ± 9.4 | 32.1 ± 9.3 | 32.3 ± 9.4 | 0.709 |
| Hospital mortality, n (%) | 116 (10.8) | 67 (12.4) | 49 (9.1) | 0.077 |
| Los hospital （day） | 8.5 (5.3, 13.5) | 8.7 (5.6, 13.7) | 8.2 (5.0, 13.1) | 0.096 |
| Los ICU (day) | 2.3 (1.3, 4.3) | 2.5 (1.4, 4.8) | 2.1 (1.3, 3.9) | 0.008 |

Abbreviations: BB, β-Blockers; HR, heart rate; MAP: Mean arterial pressure; HCT, hematocrit; HGB, hemoglobin; PLT, platelets; WBC, white blood cell count; BUN, blood urea nitrogen; MI, myocardial infarction; PVD: peripheral vascular disease; CCI: Charlson comorbidity index, SAPS II: Simplified Acute Physiology Score; Oasis: Oxford Acute Severity of Illness Score; Los: length of stay.

##

## Table S7. Baseline characteristics of patients on admission after PSM (long-acting β-blockers)

| **Variables** | **All participants**  **(n = 5324)** | **Non long-acting BB**  **(n = 2662)** | **long-acting BB**  **(n = 2662)** | ***P*-value** |
| --- | --- | --- | --- | --- |
| Gender (male %) | 2938 (55.2) | 1457 (54.7) | 1481 (55.6) | 0.508 |
| Age (year) | 73.3 ± 13.5 | 73.2 ± 13.9 | 73.3 ± 13.1 | 0.702 |
| HR (beats/min) | 83.9 ± 16.2 | 83.9 ± 16.5 | 84.0 ± 15.9 | 0.825 |
| MAP (mmHg) | 77.5 ± 9.5 | 77.4 ± 9.6 | 77.5 ± 9.3 | 0.741 |
| SpO2 | 96.6 ± 2.1 | 96.6 ± 2.1 | 96.5 ± 2.1 | 0.612 |
| HCT (%) | 30.7 ± 6.7 | 30.7 ± 6.9 | 30.7 ± 6.5 | 0.695 |
| HGB (g/L) | 10.0 ± 2.2 | 10.0 ± 2.3 | 10.0 ± 2.2 | 0.683 |
| PLT (10^9/L) | 177.0 (129.0, 237.0) | 173.0 (126.0, 235.0) | 179.0 (131.0, 239.0) | 0.016 |
| WBC (10^9/L) | 12.5 (9.0, 17.6) | 12.3 (8.6, 17.6) | 12.7 (9.4, 17.6) | 0.005 |
| Anion gap (mmol/L) | 17.0 ± 4.7 | 17.0 ± 4.7 | 17.0 ± 4.6 | 0.837 |
| BUN (mmol/L) | 30.0 (20.0, 47.0) | 30.0 (20.0, 47.8) | 29.0 (20.0, 47.0) | 0.641 |
| Calcium (mg/dL) | 8.2 ± 0.8 | 8.2 ± 0.8 | 8.2 ± 0.8 | 0.949 |
| Creatinine (mg/dL)) | 1.3 (1.0, 2.1) | 1.4 (1.0, 2.1) | 1.3 (1.0, 2.1) | 0.801 |
| Glucose (mg/dL) | 121.7 ± 43.0 | 123.2 ± 44.9 | 120.2 ± 40.8 | 0.013 |
| Sodium (mmol/L) | 139.5 ± 4.8 | 139.4 ± 5.0 | 139.6 ± 4.6 | 0.087 |
| Potassium (mmol/L) | 4.7 ± 0.9 | 4.7 ± 0.9 | 4.7 ± 0.8 | 0.533 |
| Diabetes, n (%) | 2109 (39.6) | 1055 (39.6) | 1054 (39.6) | 0.978 |
| MI, n (%) | 1751 (32.9) | 755 (28.4) | 996 (37.4) | < 0.001 |
| PVD, n (%) | 758 (14.2) | 376 (14.1) | 382 (14.4) | 0.814 |
| Dementia, n (%) | 270 ( 5.1) | 135 (5.1) | 135 (5.1) | 1 |
| Cerebrovascular disease, n (%) | 581 (10.9) | 285 (10.7) | 296 (11.1) | 0.629 |
| Chronic pulmonary disease, n (%) | 1996 (37.5) | 1023 (38.4) | 973 (36.6) | 0.157 |
| Renal disease, n (%) | 1968 (37.0) | 968 (36.4) | 1000 (37.6) | 0.364 |
| Cancer, n (%) | 669 (12.6) | 332 (12.5) | 337 (12.7) | 0.836 |
| CCI | 7.4 ± 2.5 | 7.4 ± 2.5 | 7.4 ± 2.5 | 0.957 |
| SAPS Ⅱ | 40.8 ± 13.1 | 40.8 ± 13.3 | 40.8 ± 12.9 | 0.981 |
| Oasis | 33.6 ± 9.3 | 33.6 ± 9.4 | 33.5 ± 9.2 | 0.628 |
| Hospital mortality, n (%) | 708 (13.3) | 432 (16.2) | 276 (10.4) | < 0.001 |
| Los hospital （day） | 8.8 (5.6, 14.2) | 9.1 (5.6, 14.9) | 8.5 (5.5, 13.7) | < 0.001 |
| Los ICU (day) | 2.6 (1.4, 5.0) | 2.9 (1.5, 5.4) | 2.3 (1.4, 4.3) | < 0.001 |

Abbreviations: BB, β-Blockers; HR, heart rate; MAP: Mean arterial pressure; HCT, hematocrit; HGB, hemoglobin; PLT, platelets; WBC, white blood cell count; BUN, blood urea nitrogen; MI, myocardial infarction; PVD: peripheral vascular disease; CCI: Charlson comorbidity index, SAPS II: Simplified Acute Physiology Score; Oasis: Oxford Acute Severity of Illness Score; Los: length of stay.
